# Additive Role of Immune System Infiltration and Angiogenesis in Uveal Melanoma Progression

**DOI:** 10.3390/ijms22052669

**Published:** 2021-03-06

**Authors:** Sandra García-Mulero, Maria Henar Alonso, Luis P. del Carpio, Rebeca Sanz-Pamplona, Josep M. Piulats

**Affiliations:** 1 Unit of Biomarkers and Susceptibility, Oncology Data Analytics Program (ODAP), Catalan Institute of Oncology (ICO), Oncobell Program, Bellvitge Biomedical Research Institute (IDIBELL) and CIBERESP, Hospitalet de Llobregat, 08908 Barcelona, Spain; s.garciam@idibell.cat (S.G.-M.); mhalonso@iconcologia.net (M.H.A.); 2 Department of Clinical Sciences, Faculty of Medicine and Health Sciences, University of Barcelona, 08036 Barcelona, Spain; 3 Medical Oncology Department, Catalan Institute of Cancer (ICO), IDIBELL-OncoBell, Hospitalet de Llobregat, 08908 Barcelona, Spain; lpdelcarpio@iconcologia.net; 4 Clinical Research in Solid Tumors Group (CREST), Oncobell Program, Bellvitge Biomedical Research Institute (IDIBELL) and CIBERONC, Hospitalet de Llobregat, 08908 Barcelona, Spain

**Keywords:** uveal melanoma, angiogenesis, immune system, pool analysis, prognosis, gene expression

## Abstract

Uveal melanoma (UM) is a malignant tumor that arises in the melanocytes of the uveal tract. It is the most frequent eye cancer, and despite new therapeutic approaches, prognosis is still poor, with up to 50% of patients developing metastasis with no efficient treatment options available. In contrast to cutaneous melanoma, UM is considered an “immune-cold” tumor due to the low mutational burden and the unique immunosuppressive microenvironment. To gain insight into the role of the UM microenvironment in regard to prognosis and metastatic progression, we have performed a pool analysis characterizing the UM microenvironment by using a bioinformatic approach. A variety of scores based on gene expression measuring stromal infiltration were calculated and used to assess association with prognosis. As a result, the highest immune and stromal scores were associated with poor prognosis. Specifically, stromal cells (fibroblasts and endothelial cells), T cells CD8+, natural killer (NK) cells, and macrophages M1 and M2 infiltration were associated with poor prognosis. Contrary to other tumors, lymphocytic infiltration is related to poor prognosis. Only B cells were associated with more favorable prognosis. UM samples scoring high in both angiogenesis (Angio) and antigen presentation (AP) pathways showed a poor prognosis suggesting an additive role of both functions. Almost all these tumors exhibited a chromosome 3 monosomy. Finally, an enrichment analysis showed that tumors classified as high Angio-high AP also activated metabolic pathways such as glycolysis or PI3K-AKT-MTOR. In summary, our pool analysis identified a cluster of samples with angiogenic and inflammatory phenotypes exhibiting poor prognosis and metabolic activation. Our analysis showed robust results replicated in a pool analysis merging different datasets from different analytic platforms.

## 1. Introduction

Uveal melanoma (UM) is a malignant tumor arising at the melanocytes of the uveal tract [1]. It is the most frequent cancer in the eye, and is considered a rare tumor (10 cases per million incidence in Europe) [2]. Prognosis in UM is poor, with median overall survival (OS) of less than one year in most cases, and up to 50% of patients developing metastasis (M1), mostly in the liver. Currently, metastatic UM (MUM) does not have an effective standard treatment available and survival rates have not improved in the last decades [1,3,4,5]. Recent meta-analysis reviews on progression-free survival (PFS) and overall survival (OS) from different clinical trials have shown that none of the different novel treatments carried out in recent years has improved the prognosis of UM patients, reinforcing the need for further research.

Immunotherapy has shown extraordinary results on cutaneous melanoma (CM). Monoclonal and combined therapies with anti-programmed death ligand 1 (PDL1) and anti-cytotoxic T-lymphocyte antigen-4 (CTLA-4) checkpoint inhibitors are already a standard therapy for CM. However, these results have not been reproduced in UM [6,7], which differs from CM at the genetic and molecular level and should be treated with specific treatments [8]. One important difference is the tumor mutational burden (TMB), which is very high in CM, which generates a great amount of neoantigens that renders high immunogenicity and attracts T CD8+ lymphocytes. By contrast, UM has a low TMB, and therefore is considered a tumor with low antigenicity [9].

Tumors with microsatellite instability (MSI) phenotype or harboring mutations in mismatch repair (MMR) genes are highly mutated. A recent study analyzing the frequency of MMR genes in three independent cohorts of UM patients showed that mutations in these genes were extremely rare [10]. Also explaining the low TMB, a work by Cross et al. analyzing microsatellite instability in UM demonstrated that, in contrast to CM, MSI not occurring in UM [11]. A study by Johansson et al. analyzing 103 UM by whole-genome sequencing from different sites of the uveal tract demonstrated that only patients with tumors located in the iris showed high TMB. This phenotype is associated with ultraviolet radiation signature, common in CM. However, only 8 out of 103 analyzed tumors were located in the iris [12]. Furthermore, UM is located in an immune-privileged organ with an immunosuppressive microenvironment, protected by the blood–ocular barrier, and an absence of lymphatic vessels that prevent the traffic of immune cells to the eye [13].

Molecular profiling has provided a new perspective of the biology of UM. The Cancer Genome Atlas (TCGA) recently performed the analysis of 80 UM primary tumors and identified four different molecular subtypes [14]. Molecular subgroups 1 and 2 are associated with disomy of chromosome 3 (D3) and better prognosis, whereas subgroups 3 and 4 are associated with monosomy of chromosome 3 (M3) and have worse prognosis. The immune profiling of TCGA-UM analysis is scarce and limited to the association of M3 tumors with higher levels of CD8+ T cells, Interferon gamma signaling, and immune suppressor factors. Therefore, the immune microenvironment of UM seems to be related to the genomic alterations, mostly to mutations in BAP1 gene, rather than to response to immune signaling [15]. In this regard, it has been recently described that UM tumors harboring mutations in BAP1 gene showed upregulation of several genes associated with suppressive immune responses [16].

Dissemination in UM is hematogenous, suggesting an important role of tumor angiogenesis (the development of new blood vessels) in tumor growth and metastasis. Indeed, we have previously shown that enrichment in pro-angiogenic factors was related to worse prognosis in UM but not in CM [17]. Angiogenesis is directly associated with immune evasion and resistance to immunotherapy by suppressing dendritic cell maturation, inhibiting T-cell effector response and recruiting myeloid derived suppressor cells. Thus, therapeutic strategies combining immunotherapy with anti-angiogenic factors could modulate the tumor microenvironment to make it more susceptible to the immune checkpoint inhibitors.

In this study, we perform a bioinformatics analysis using public gene expression data in order to perform an in-depth characterization of the tumor microenvironment of UM primary tumors and assess its association with prognosis.

## 2. Results

### 2.1. Clinical Description

A total of 213 primary UM patients from 5 datasets were included in the meta-analysis, described in Table 1. The median age was 62.3, with 41.3% female and 58.7% males. Up to 56% of patients had recurred with a median disease-free survival (DFS) of 38.6 months. Differences between the different datasets were evaluated for continuous variables (Kruskal–Wallis test) and categorical variables (Chi-squared test of proportions). No differences were found for age, sex and recurrence status, while strong differences between datasets were found on chromosome 3 status, cell type and DFS. Due to the differences between datasets for DFS, all survival analyses were performed stratified by dataset.

### 2.2. Stromal and Immune Cell Infiltration Is Associated with Poor Prognosis in Uveal Melanoma (UM)

**Figure 1:**
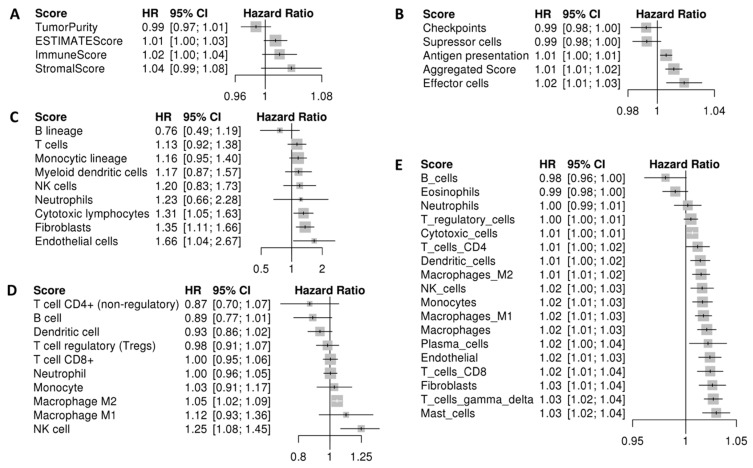
Forest plot showing survival pool analysis using immune and stromal infiltration scores. Horizontal bars indicate the 95% confidence intervals (CI) of the hazard ratio (HR). Each score was evaluated individually and ordered based on the summary effect. Univariate Cox HR analysis were performed using the disease-free survival time. (**A**) HR based on the ESTIMATE scores. (**B**) HR based on the Immunophenoscore individual scores and Aggregated score. (**C**) HR based on immune cell infiltrate scores from MCP-counter. (**D**) HR based on the immune infiltrates from Quantiseq analysis. (**E**) HR based on markers of immune infiltrates from Consensus^TME^.

A variety of scores based on gene expression measuring stromal and immune cell infiltration were calculated and used to assess association with prognosis. First, we used the ESTIMATE (Estimation of Stromal and Immune Cells in Malignant Tumor Tissues Using Expression Data) tool to measure tumor purity and immune cell/stromal infiltration. The four resulting scores were used as global indicators since the tool does not deconvolute between different cell lineages. The tumor purity score had a trend toward better prognosis, although it was not significant (HR = 0.99 [0.97–1.01]). On the contrary, The ESTIMATE score, which is a measure of non-tumoral cell infiltration was associated with worse prognosis (HR = 1.01 [1–1.03]). The ESTIMATE score was calculated based on both immune and stromal scores, both associated with bad prognosis (immune score HR = 1.02 [1–1.05]; stromal score HR = 1.04 [0.99–1.08]), Figure 1A. Thus, highly infiltrated tumors (with both stromal and immune cells) showed a poor prognosis. In other words, the more tumor purity the better the prognosis. Table S1 shows the ESTIMATE scores in each sample.

Next, immunophenoscore (IPS) scores were used as an indicator of immune system activation (Figure 1B). The aggregated score, a composite score measuring the overall immunogenicity of a tumor, was associated with poor prognosis (HR = 1.01 [1.01–1.02]). Interestingly, when individual scores were interrogated, effector cells (HR = 1.02 [1.01–1.03]) and antigen presentation (HR = 1.01 [1–1.01]) scores were associated with poor prognosis. On the contrary, checkpoints markers (HR = 0.99 [0.98–1]) and suppressor cell scores (HR = 0.99 [0.98–1]) were associated with better prognosis (Figure 1B). Forest plots showing separated ESTIMATE and IPS analysis for each dataset are available in Figure S1. In summary, immunogenic tumors (those scoring higher in IPS) were associated with poor prognosis. Moreover, if samples were divided between high and low infiltrated according to the ESTIMATE score, those included in the high category scored higher in Antigen presentation and effector cells (Figure S2A). Also, they show a trend toward poor prognosis (Log-rank *p*-value = 0.07) (Figure S2B).

Finally, since ESTIMATE only performs a global estimation of non-tumoral cell infiltration, a cell-type detailed analysis was done using quantification methods based on gene expression. A general trend towards infiltration association with poor prognosis was observed. However, exceptions in several cell lineages emerged in the analysis (Figure 1C–E). The MCP-counter (Microenvironment Cell Populations-counter) method selected B cells as the only cells associated with better prognosis (HR = 0.76 [0.49–1.19]). By contrast, cytotoxic lymphocytes (HR = 1.31 [1.05–1.63]), fibroblasts (HR = 1.35 [1.11–1.66]), and endothelial cells (HR = 1.66 [1.04–2.67]), were significantly associated with poor prognosis (Figure 1C). The Quantiseq method, measuring additional cell lineages, showed CD4 T-cells (HR = 0.87 [0.70–1.07]), B-cells (HR = 0.89 [0.77–1.01]), and dendritic cells (HR = 0.93 [0.86–1.02]), were associated with better prognosis. On the contrary, macrophages M1 (HR = 1.12 [0.93–1.36]), macrophages M2 (HR = 1.05 [1.02–1.09]), and NK cells (HR = 1.25 [1.08–1.45]) were associated with poor prognosis. Additionally, the Consensus^TME^ (Consensus Tumor Microenvironment) method also selected B-cells (HR = 0.98 [0.96–1]), as good prognosis biomarkers along with eosinophils (HR = 0.99 [0.98–1]). Inconsistent results were found across different quantification methods when dendritic cells (DC) were interrogated. Since this is a heterogenous group of cells, we use additional signatures to discriminate between immature DC (iDCs) and activated DC (aDCs). However, we did not observe an association between iDCs and/or aDCs with prognosis (Figure S3). The genes included in all signatures used in the analysis are listed in Table S2.

In summary, all methods indicated that B cells are associated with better prognosis. By contrast, stromal cells (fibroblasts and endothelial cells), T cells CD8+, NK cells, and macrophages M1 and M2 were associated with poor prognosis in at least two out of the three methods evaluated. Interestingly, B cells act as APCs through HLA class II whereas NK and CD8+ T cells destroy cells not expressing HLA class I or cells presenting antigens through HLA class I, respectively. Since the later are inflammation-related pathways classically associated with better prognosis in other solid tumors, we speculated that UM is a divergent type of tumor in this regard.

**Figure 2:**
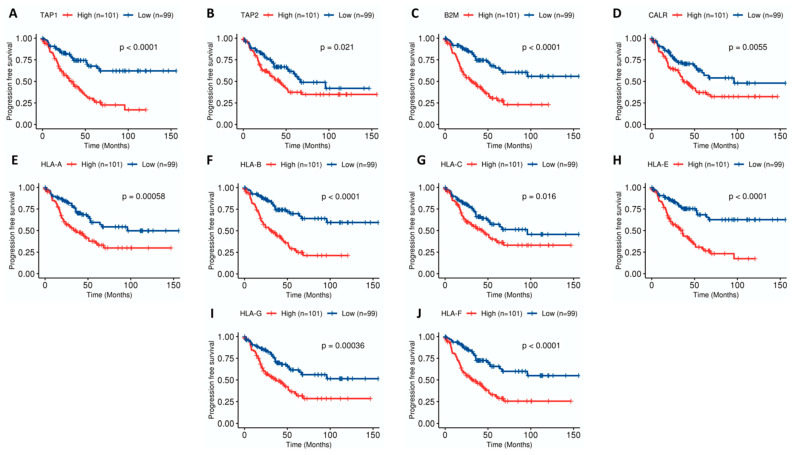
Kaplan-Meier survival curves with genes related to antigen processing and presentation machinery in uveal melanoma (UM) samples (*n* = 200). Genes included are (**A**) tapasin-1 (TAP-1), (**B**) TAP-2, (**C**) beta-2-microglobulin (B2M), (**D**) calreticulin (CALR), (**E**) human leukocyte antigen-A (HLA-A), (**F**) HLA-B, (**G**) HLA-C, (**H**) HLA-E, (**I**) HLA-G, (**J**) HLA-F. Patients were divided into High and Low groups by the median value within each dataset and joined afterwards. *p*-values of Log-rank tests are indicated. High expression is painted in red whereas low expression is painted in blue.

Thus, to see whether antigen presentation genes were prognosis biomarkers in UM, a survival analysis was done. Kaplan-Meier plots in Figure 2 showed that tumors showing high expression levels of genes related to the antigen presentation pathway were associated with poor prognosis. Tapasin 1 (TAP1) (Figure 2A), beta-2-microglobulin (B2M) (Figure 2C), human leukocyte antigen-B (HLA-B) (Figure 2F), HLA-E (Figure 2H) and HLA-F (Figure 2J) were the more significant genes with Log-rank *p*-values < 0.0001. Regarding cytotoxicity, tumors showing high expression of CD8A, GZMA and PRF1 were associated with poor prognosis (Figure S4). 

Mutational data was only available in the TCGA dataset. To see if the number of mutations was associated with prognosis, we calculated the tumor mutational burden (TMB). As expected in a cold tumor, only a median of 16 mutations per sample were found. TMB was not associated with prognosis.

### 2.3. Combination of Angiogenesis and Antigen Presentation Confers Poor Prognosis

Results showed that endothelial cells, fibroblasts (stromal cells) and immune cells (specifically cytotoxic ones) were associated with poor prognosis. Across these three microenvironmental factors, overlapping genes are scarce providing strength to the analysis.

Only one gene (HIF1A) has been found in common between fibroblasts and Angiogenesis signature. Consensus^TME^ signatures measuring immune cell infiltration overlap with angiogenesis signature in: B cells (PRKCB), monocytes (HIF1A and ARNT), Macrophages M2 (FLT1), T cells CD4+ (EIF2B5 and PLCG1), T cells CD8+ (FLT4 and PLCG1).

**Figure 3:**
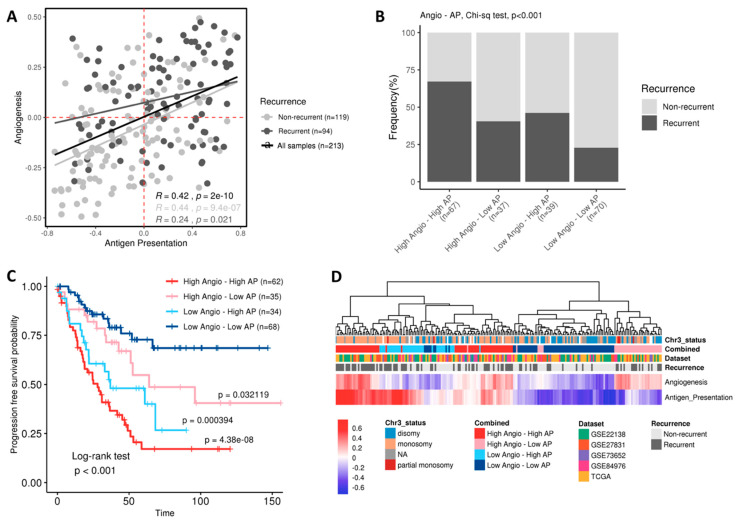
Combination of enrichment scores from angiogenesis (Angio) and antigen presentation (AP) signatures. (**A**) Correlation plot of Angio and AP signatures, colored by recurrent (light grey) and non-recurrent (dark grey) status. Black line represent correlation for all samples, regardless of recurrence status. The Spearman correlation was calculated and regression lines correlation scores and *p*-values were added for each recurrence status and for all the samples. Dashed lines indicate the cut-off used for generating the four groups. (**B**) Bar plot showing the frequency of recurrent samples in the different groups of Angio-AP combinations. (**C**) Kaplan–Meier survival curves of the four groups from combination of Angio and AP scores. Log-rank *p*-value is indicated. Cox proportional hazard ratios test between the Low Angio-Low AP reference group and the other three groups was calculated, and *p*-values are also indicated. (**D**) Hierarchical clustering of all UM samples (*n* = 213) using the Angio and AP scores. Bars on top represent chromosome 3 status, dataset, recurrence and combined score.

Apart from fibroblasts, endothelial cell infiltration was strongly associated with relapse; in agreement with previous results in the group. Thus, we wonder if these two features were additive and confers a particularly aggressive phenotype. To do this, we used angiogenesis (Angio) and antigen presentation pathways (AP) as reporter ones. Antigen presentation pathway was used as a surrogate of immune system activation. First, we assessed if a correlation between these two features existed. A statistically significant but moderate correlation was found when all samples were taken together (R = 0.42, *p* = 2 × 10^−10^, black line in the Figure 3A) and also when stratifying between relapsing (R = 0.24, *p* = 0.021, dark grey line in Figure 3A) and non-relapsing patients (R = 0.44, *p* = 9.4 × 10^−7^, light grey in Figure 3A). Next, we classified the samples based in the combination of the scores, obtaining four groups (High Angio-High AP, High Angio-Low AP, Low Angio-High AP, Low Angio-Low AP). As can also be seen in Figure 3A, the bar plot in Figure 3B showed that High Angio-High AP group includes the high number of recurrent samples, whereas Low Angio-Low AP group includes the higher number of non-recurrent. Phenotypes High Angio-Low AP and Low Angio-High AP exhibited an intermediate, similar numbers of recurrent samples (Chi-squared test, *p* = 4.5 × 10^−6^).

Indeed, a survival analysis shows strong differences in DFS probability between the four groups (Log-rank test, *p* < 0.0001), being the High Angio-High AP the group with worse prognosis in opposition to Low Angio-Low AP group (*p*-value = 4.4 × 10^−8^) which is the group showing better survival (Figure 3C). Because intermediate phenotypes (High Angio-Low AP and Low Angio-High AP) have intermediate survival probability and those tumors classified as High Angio-High AP were more prone to metastasize, we think these two features had an additive role in prognosis. We tested the hypothesis that differences in recurrence time could exist between group of samples, being Low Angio-Low AP patients those relapsing later. Although no statistical differences were found, Figure S5 showed a trend towards High Angio-High AP tumors as the earlier relapsing ones.

Finally, we performed an unsupervised hierarchical clustering using Angio and AP scores that reflected the four groups previously described, as expected. The High Angio-Low AP and Low Angio-Low AP were separated clusters. A mixture in the dendrogram was observed between Low Angio-High Ap and High Angio-High AP groups (Figure 3D). It is worth mentioning that there is an enrichment in samples harboring disomy in chromosome 3 in the Low Angio-Low AP cluster. On the contrary, almost all High Angio-High Ap exhibited a chromosome 3 monosomy.

**Figure 4:**
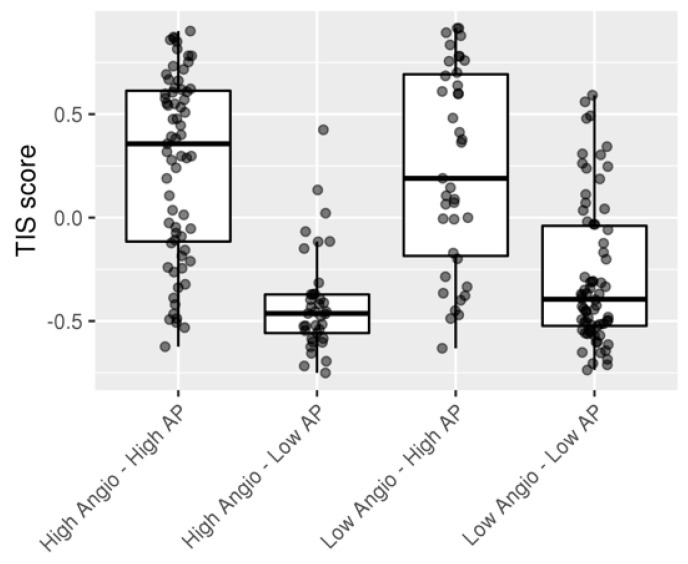
Boxplot showing T-cell inflammatory signature (TIS) score across the four Angio-AP combination groups.

Next, we consider whether the poor prognosis group might be susceptible to treatment with immunotherapy. To answer this question, samples were scored using a genetic profile reported as a good predictor of clinical response to anti-PDL1 pembrolizumab (T-cell inflammatory signature (TIS) score). As expected, tumors with inflammatory phenotype High Angio-High AP showed high TIS score values. Also, Low Angio-Low AP tumors showed very low values. However, it is interesting to note that there was High Angio-High AP samples with low TIS score. On the contrary, a group of Low Angio-High AP samples showed high TIS score thus was susceptible to be treated with immunotherapy (Figure 4).

Finally, we wonder if fibroblasts (Fibro) also have an additive effect on prognosis, along with AP. First, we assessed correlation between fibroblasts and endothelial cells infiltration finding a strong correlation (R = 0.7, Figure S6A). Therefore, we expected similar results with Angio/AP than with Fibro/AP. Indeed, the correlation between Fibro/AP is very similar to correlation between Angio/AP (statistically significant but moderate R = 0.44, Figure S6B). When tumors were stratified in four groups (High AP-High Fibro, High Ap-Low Fibro, Low AP-High Fibro and Low AP-Low Fibro), tumors in Low AP-Low Fibro showed the better prognosis (Figure S6C).

### 2.4. Metabolic and Tyrosine Kinase Pathways Are Activated in Poor-Prognosis Tumors

**Figure 5:**
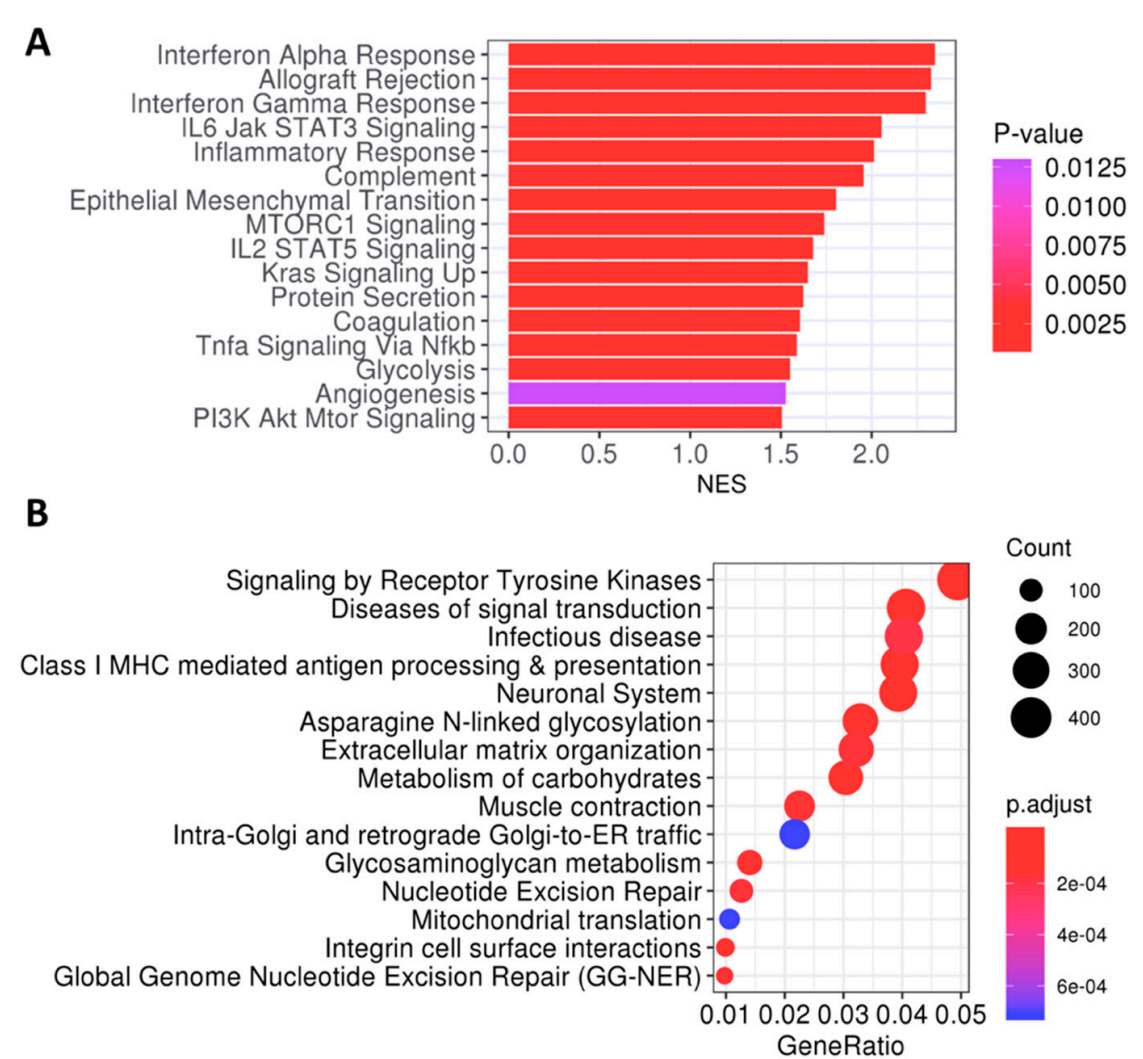
Functional analysis of recurrent tumors within phenotypes “High Angio-High AP” (*n* = 45) versus “Low Angio-Low AP” (*n* = 16). (**A**) Bar plot of enriched Hallmarks from pre-ranked GSEA analysis, ordered by NES and colored by *p*-values. (**B**) Dot plot of enriched pathways from DEGs in “High Angio-High AP” phenotype, colored by adjusted *p*-values. GeneRatio correspond to the frequency of genes from the gene set in the list of DEGs. Count represents the total number of genes. GSEA: Gene Set Enrichment Analysis, NES: Normalized Enrichment Score, DEGs: Differentially Expressed Genes.

In order to deepen our knowledge of the biological behavior of tumors with poor prognosis, a functional analysis comparing relapsing tumors between extreme phenotypes was conducted. As expected, the enrichment analysis (Figure 5A) showed an angiogenesis and inflammatory response in High AP-High Angio tumors, along with immune-related pathways such as complement, tumor necrosis factor alpha (TNF-α) and interleukin-2 (IL2) signaling. However, other non-infiltration-related pathways emerged, such as glycolysis, epithelial-mesenchymal transition, KRAS signaling, mTORC1 signaling, and PI3K-AKT-MTOR signaling. This suggested a crosstalk between immune infiltration, angiogenesis and metabolic pathways. Similar results were achieved when the permuting-labels method was utilized instead of the pre-ranked gene set enrichment analysis (GSEA). An alternative method, using the most differentially expressed genes, reported signal transduction pathways and metabolisms-related pathways as the most significant ones, as well as the antigen presentation pathway (Figure 5B).

## 3. Discussion

**Figure 6:**
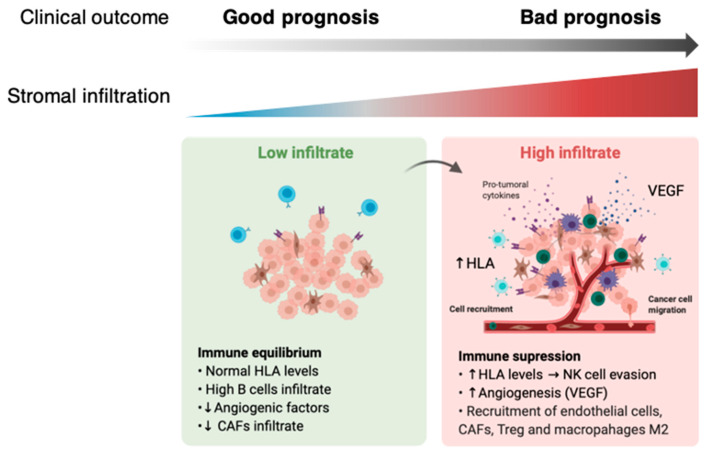
Hypothesis. Our results suggest a combinatory function of angiogenesis and inflammation in UM tumors. Infiltration of macrophages, fibroblasts and natural killer (NK) cells is associated with poor prognosis, whereas infiltration of B cells is associated with good prognosis. HLA over-expression could trigger a mechanism to evade the NK-cells mediated attack. This immunosuppressive environment, together with the high levels of stromal activity and checkpoints blockade, allows UM cells to disseminate and metastasize. New therapeutic strategies such as, combining immune checkpoint inhibitors (anti-programmed death ligand 1 (PDL1) or anti-cytotoxic T-lymphocyte antigen-4 (CTLA4)) with anti-angiogenic targeted therapy (anti-vascular endothelial growth factor (VEGF)) could improve patients’ response. Figure created with BioRender.com.

To show the importance of inflammation and other stromal cells, we have performed a pool analysis of 5 datasets containing prognostic, and transcriptomic information from 213 primary UM samples available in the literature [14,18,19,20,21]. We have identified a cluster of samples with angiogenic and inflammatory phenotypes exhibiting poor prognosis (Figure 6). Contrary to what is found in other tumors, lymphocytic infiltration is related to poor prognosis. In a similar vein, a study by Luo and Ma [22] in UM also associated CD8 lymphocytic infiltration with poor prognosis (univariate analysis), and B-cell infiltration with better prognosis (multivariate analysis). This association between inflammation and poor prognosis in uveal melanoma has already been described in individual studies [14,23,24,25].

Tumor infiltrating lymphocytes (TILs) in primary UM are mainly CD8+ cytotoxic T cells and were present in all 43 cases analyzed by Bronkhorst et al. [23]. In addition, CD4+ T-helper cells could also be found in 91% of the samples, and approximately half of these were FoxP3+ regulatory T cells. It is also noteworthy that a well-characterized prognostic factor in UM, such as chromosome 3 monosomy, seems to be strongly correlated with larger lymphocytic infiltrate. The key question here is why TILs lead to poor prognosis. One of the hypotheses is that metastatic dissemination is required to create a T-cell response due to the peculiar immune ocular characteristics [25]. If that hypothesis is true, only the UM that disseminates outside the eye should have TILs in the primary tumor. The immunosuppressive microenvironment of the primary site, along with inhibitory characteristics displayed by UM cells, would render this infiltrate non-effective when it comes to immune-surveillance. Another, more intriguing, possibility is that TILs not only fail to eliminate tumor cells but also help tumor growth [26]. Many examples demonstrate that inflammation can promote proliferation and survival of cancer cells [27]. Activated TILs would produce inflammatory mediators, generating a cancer-related inflammatory microenvironment. The cell-type detailed analysis performed using quantification methods based on gene expression found cytotoxic lymphocytes, macro-phages and NK cells associated with poor prognosis in at least two of the three methods evaluated. On the other hand, B cells were found to be correlated with better prognosis with all bioinformatic tools tested.

There are no previous reports associating B cells with better prognosis in uveal melanoma, but recently different studies have associated the presence of B cells with survival and immunotherapy response in different tumors [28,29,30].

The cell-detailed analysis also revealed fibroblasts and endothelial cell signatures associated with poor prognosis. UM arises in one of the most capillary-rich tissues of the body and disseminates hematogenously. Highly vascularized UM tumors are more aggressive and indicate a worse prognosis. Recently, we have shown that primary UM included in the TCGA that relapses systemically shows a much higher angiogenesis enrichment score than non-relapsed patients [17]. Differences in disease-free survival (DFS) when comparing high vs. low angiogenesis enrichment scores were statistically significant in UM patients but did not show significance when we compared signature high vs. low using primary tumors included in the CM dataset from the TCGA.

We next sought to study if there was association between T-cell activation and angiogenesis signatures. We identified a group of patients with extremely poor prognosis characterized by high levels of activation of both signatures and 82.9% 5-year relapse rate. On the other hand, tumors with low activation showed good prognosis with only 31.5% of patients relapsing at 5 years. VEGF, along with other angiogenic factors, plays a crucial role in modulating the immune system and fostering an immunosuppressive microenvironment by directly suppressing dendritic cell maturation, inhibiting T-cells by enhancing PD-1 and other inhibitory checkpoints, disrupting the normal differentiation of hematopoietic precursor cells, and recruiting immunosuppressive cells such as T-cells and myeloid derived suppressor cells [31,32]. Thus, a pro-angiogenic environment could be a reason why immunotherapy with checkpoint inhibitors has not been very effective in metastatic uveal melanoma compared to other tumors. Angiogenesis activation has been a hallmark of tumors resistant to checkpoint inhibitors and is associated with more immune-suppressed stroma in different cancers probably due to the close relationship between aberrant cancer angiogenesis and immunosuppression [33,34].

Several antiangiogenic drugs have been used to treat MUM [17]. It is difficult to reach a full conclusion because most of the trials are small and lack a comparator arm, but from the results we can assume that although no objective responses are seen, clinical trials with antiangiogenic drugs usually show slightly higher PFS and OS than clinical trials with conventional chemotherapy. Based on these observations, it would be of special interest to test the activity of antiangiogenic drugs combined with checkpoint inhibitors and see if we can reproduce results observed in other diseases where checkpoint inhibitors in monotherapy do not work such as endometrial cancer. Interestingly, hepatocellular carcinoma, another disease with liver involvement, has become a target for this combination strategy after the IMbrave150 (Atezolizumab plus Bevacizumab in Unresectable Hepatocellular Carcinoma) study showed the superiority of atezolizumab and bevacizumab vs. sorafenib in terms of OS [35].

In order to find other weaknesses that could arise due to therapeutic interventions, we compared primary UM tumors with high angiogenic and antigen presentation activation, with tumors with low activation of both pathways. Interestingly, glycolysis and the PI3K-AKT-MTOR pathways were among the non-infiltration-related pathways. These metabolic pathways have already been linked to immune-resistance to checkpoint inhibitors in different studies [34,36,37,38]. Interestingly, a recent study has shown that UM is among the tumors with the highest oxidative phosphorylation gene expression and correlates with prognosis in primary UM [39]. Our group has recently made the same observation in MUM patients [40]. We evaluated glucose metabolism in liver metastasis using positron emission tomography (PET-CT) with [18F]-fluorodeoxyglucose (FDG). Increased metabolic activity in liver metastases was found to be an independent predictor of overall survival even in patients with small lesions (M1a).

Our analysis showed robust results replicated in a pool analysis merging different datasets from different analytic platforms. However, this study has limitations. Because it is an in-silico analysis using public data, we have no control over initial stages of the analysis such as sample selection or RNA extraction. Transcriptomic data in each dataset has been analyzed and normalized separately. Also, clinical information is scarce. Specifically, anti-tumoral treatments could affect stromal and immune infiltration. Unfortunately, therapy information was not available and/or detailed. Thus, further experimental validation is needed in order to validate the hypothesis in Figure 6.

## 4. Materials and Methods

### 4.1. Patients and Samples

Gene expression, mutations, and clinical data from 80 UM primary tumor samples from the TCGA-UM dataset were collected from the cBioPortal. RNA-seq was downloaded in fragments per kilobase per million (FPKM), then converted to log2 scale. Also, a total of 133 UM primary samples analyzed using microarrays were downloaded from the Gene Expression Omnibus (GEO) repository; accession numbers GSE27831 (*n* = 29) [18], GSE22168 (*n* = 63) [19], GSE73652 (*n* = 13) [20], GSE84976 (*n* = 28) [21]. Gene expression data from GEO was log2 scaled. To control for the batch effect, an adjustment was performed using Combat function from R package “sva”. All datasets include information of progression-free survival (PFS), except GSE73652 which only includes the recurrence status. Table S3 includes a detailed description of the datasets.

### 4.2. Microenvironment Characterization

Gene expression data was used to characterize the immune microenvironment of samples, using a variety of bioinformatics tools. The immunophenoscore (IPS) function was used to measure the immune state of the samples [41]. IPS uses a number of markers of immune response or immune toleration to quantify four different immune phenotypes in a tumor sample (Antigen Presentation, Effector Cells, Suppressor Cells and Checkpoint markers). It also generates an aggregated z-score summarizing the global immune state of the samples. Then, the ESTIMATE R package was used to predict the purity from the samples. ESTIMATE is a tool that predicts the tumor purity and infiltrating stromal/immune cells from gene signatures [42]. ESTIMATE calculates four scores. The Stromal score and the Immune score were calculated using gene expression signatures. Then, a combination of both generates the ESTIMATE score, a measure of the global infiltration of non-tumoral cells in a sample. Finally, the ESTIMATE score is used to calculate the score called Tumor purity, a measure of the number of tumor cells in a sample that are inversely correlated with the ESTIMATE score. The higher the ESTIMATE score, the lower the Tumor purity score.

Three different methodologies of quantification were used; MCP-counter, Quantiseq, and Consensus^TME^. We have followed the recommendations by Strum et al. [43] and used three different methods to obtain robust results. However, each methodology analyzes a different number of cell types and with different levels of detail that can generate ambiguity when comparing results. The Immunedeconv R package was used to infer cell infiltration using MCP-counter and Quantiseq methods [43]. MCP-counter is a method based on marker genes that quantifies the relative fraction of 10 cell types, including two stromal cell types. Quantiseq is a deconvolution method that infers the absolute fraction and shows a more detailed picture of the immune cell subtypes. Finally, a list of the total 18 gene markers from Consensus^TME^ was used to perform the enrichment analysis with gene set variation analysis (GSVA) [44]. All analyses were performed independently for the different datasets and resultant scores matrices were joined for further analysis.

In addition, we have searched for gene expression signatures discriminating between dendritic cells. Specifically, immature dendritic cells (iDC) and activated dendritic cells (aDC) were extracted from Tamborero et al. [34].

### 4.3. TMB (Tumor Mutational Burden)

Mutational data was only available in the TCGA data and were used to calculate tumor mutational burden (TMB) per sample.

### 4.4. Angiogenesis and Antigen Presentation Enrichment Analysis

Angiogenesis (Angio) and antigen presentation (AP) gene sets were manually selected from the curated gene set collection of the Molecular Signatures Database (MSigDB) (BIOCARTA_VEGF_PATHWAY and REACTOME_ANTIGEN_PRESENTATION, respectively). The GSVA R packages were used to perform Gene Set Variation Analysis [45]. This function performs a non-parametric, unsupervised analysis for estimating variation of the given gene sets through the samples in the expression matrix, returning an enrichment score for each sample. The GSVA function was performed with 1000 bootstraps and arguments as default. GSVA was performed independently on the different datasets and resultant matrices were joined for further analysis. The association between the two scores was evaluated with the Spearman correlation test for all samples, recurrent and non-recurrent. Samples were divided into “High” and “Low” groups for each score, with a zero value as cut-off. Next, samples were divided into four groups based on the combination of the two scores (High Angio-High AP; High Angio-Low AP; Low Angio-High AP; Low Angio-Low AP). The frequency of recurrence between the four groups was evaluated with a Chi-squared test.

### 4.5. Hierarchical Clustering

Samples were clustered by agglomerative hierarchical clustering on the basis of the GSVA enrichment scores on the two selected gene sets. Sample distances were computed via the R function “dist”, with Euclidean distance. Next, the “hclust” function generated a clustering from the distances, with the “average” linkage method. Finally, a heatmap was plotted and a dendrogram was drawn for distance-tree visualization purposes.

### 4.6. Functional Analysis

To identify enrichment in specific cellular functions and pathways, a GSEA was performed comparing the recurrent samples from extreme phenotypes (‘High Angio-High AP’ vs. ‘Low Angio-Low AP’) [46]. GSEA analysis was performed with the clusterProfiler R package. Gene sets Hallmarks and Canonical pathways from MSigDB were interrogated. Samples were scored with the GSVA method using the T-cell inflammatory (TIS) signature [47].

### 4.7. Statistical Methods

All statistical analyses were performed with R version 3.5.0 (R Foundation for Statistical Computing, Vienna, Austria). For homogenization of methods, all comparisons between continuous variables were analyzed using non-parametric tests (Wilcoxon test and Kruskal–Wallis test). For all tests applied, differences were considered statistically significant when *p*-value < 0.05. The Cox proportional hazard regression model was used to assess the prognostic effect of the different scores. Cox analysis was performed independently for the different cohorts. A random-effects model was used to summarize the effect for each of the different scores with the “metagen” function from R package meta. This function performs a meta-analysis based on hazard ratio estimates and their standard errors. The overall effect is calculated with the inverse variance method. The results were plotted in a forest plot. For the pool analysis, all samples were joined and a Cox regression model stratified by dataset was performed. Results were summarized and plotted with a forest plot. Survival probabilities were plotted with the Kaplan–Meier method, and the Log-rank test was used to compare the survival proportions among different groups. For categorical variables, samples were divided into “High” and “Low” groups based on the cut-off points (zero value for scores, median value for gene expression).

## Figures and Tables

**Table 1:** Baseline characteristics of samples included in the analysis by dataset. *p*-values for categorical variables indicate results from Chi-Squared Tests. *p*-values for continuous variables indicate results from Kruskal tests. DFS: disease-free survival.

Variable	Entire Cohort *n* = 213	GSE22138 *n* = 63	GSE27831 *n* = 29	GSE73652 *n* = 13	GSE84976 *n* = 28	TCGA *n* = 80	*p*-Value
Age	62.3	61.0	66.0		61.6	62.2	0.411
Sex							0.801
Female	71 (41.3%)	24 (38.1%)	12 (41.4%)	0 (0.0%)	0 (0.0%)	35 (43.8%)	
Male	101 (58.7%)	39 (61.9%)	17 (58.6%)	0 (0.0%)	0 (0.00%)	45 (56.2%)	
NA	41	0 (0.0%)	0 (0.0%)	13 (100%)	28 (100%)	0 (0.0%)	
Chr 3 status							<0.001
Disomy	64 (32.2%)	18 (28.6%)	11 (37.9%)	0 (0.0%)	14 (50.0%)	21 (26.2%)	
Partial monosomy	5 (2.5%)	5 (7.94%)	0 (0.0%)	0 (0.0%)	0 (0.0%)	0 (0.0%)	
Monosomy	94 (47.2%)	32 (50.8%)	17 (58.6%)	0 (0.0%)	14 (50.0%)	31 (38.8%)	
NA	36 (18.1%)	8 (12.7%)	1 (3.5%)	13 (100%)	0 (0.0%)	28 (35.0%)	
Cell type							<0.001
Epithelioid	40 (18.7%)	21 (33.3%)	6 (20.7%)	0 (0.0%)	0 (0.0%)	13 (16.2%)	
Mixed	72 (33.8%)	23 (36.5%)	12 (41.4%)	0 (0.0%)	0 (0.0%)	37 (46.2%)	
Spindle	39 (18.3%)	0 (0.0%)	9 (31%)	0 (0.0%)	0 (0.0%)	30 (37.5%)	
NA	60 (28.2%)	19 (30.2%)	0 (0.0%)	13 (100%)	28 (100%)	0 (0.0%)	
Recurrence							0.245
Non-recurrent	119 (55.9%)	28 (44.4%)	18 (62.1%)	8 (61.5%)	15 (53.6%)	50 (62.5%)	
Recurrent	94 (44.1%)	35 (55.6%)	11 (37.9%)	5 (38.5%)	13 (46.4%)	30 (37.5%)	
DFS Months	38.6	41.1	37.2		77.8	23.2	<0.001

## Data Availability

Publicly available datasets were analyzed in this study. All R code used for the analyses are openly available for reproducibility of the results in a GitHub repository at https://github.com/odap-ubs/uveal-pool-analysis.

## Supplementary Materials

The following are available online at https://www.mdpi.com/1422-0067/22/5/2669/s1. Figure S1: Meta-analysis of immune markers by dataset for the different scores of ESTIMATE (A) and Immunophenoscore (B). Figure S2: A. Boxplots of scores of antigen presentation, effector cells, suppressor cells and checkpoint markers for High (*n* = 106) and Low (*n* = 107) Immune groups. B. Kaplan-Meier survival curves for High and Low groups. Patients were divided into High and Low groups by the median value of the ESTIMATE score. Figure S3: Meta-analysis of dendritic cell markers by dataset for Activated dendritic cells (aDCs) (A) and Immature dendritic cells (iDCs) (B). Figure S4: Kaplan-Meier survival curves for cytotoxic markers CD8A (A), GZMA (B) and PRF1 (C). Patients were divided by the median expression value for each dataset and joined afterwards. Figure S5: Boxplots showing time to recurrence by Angio-AP groups. Figure S6: (A) Fibroblasts and endothelial cells correlation plot (Spearman correlation). (B) Correlation plot between Fibroblasts and Antigen Presentation signatures colored by recurrent (light grey) and non-recurrent (dark grey) status. Black line represents correlation for all samples, regardless of recurrence status. Spearman correlation is indicated (C) Kaplan-Meier survival curves for the four groups combining Fibroblasts and AP scores. Table S1: Resultant scores of the ESTIMATE method and immune groups generated from the ESTIMATE score. Table S2: Gene signatures of the different methods used in the analysis. Table S3: Detailed clinical and molecular information for each dataset.

## Abbreviations

aDC	activated dendritic cell
Angio	Angiogenesis
AP	Antigen Presentation
B2M	Beta-2-Microglobulin
BAP1	BRCA1 Associated Protein 1
CALR	Calreticulin
CI	Confidence Interval
CM	Cutaneous Melanoma
CTLA-4	Cytotoxic T-Lymphocyte Antigen-4
D3	Disomy of Chromosome 3
DC	dendritic cell
DEG	Differentially Expressed Genes
DFS	Disease Free Survival
FPKM	Fragments per Kilobase per Million
GEO	Gene Expression Omnibus
GSEA	Gene Set Enrichment Analysis
GSVA	Gene Set Variation Analysis
GZMA	Granzyme A
HLA	Human Leukocyte Antigen
HR	Hazard Ratio
iDC	immature dendritic cell
IL2	Interleukin-2
IPS	Immunophenoscore
KM	Kaplan-Meier
M1	liver metastasis
M1a	small lesion liver metastasis
M3	Monosomy of Chromosome 3
MSigDB	Molecular Signatures Database
mTORC1	Mammalian target of rapamycin complex 1
MUM	Metastatic Uveal Melanoma
NES	Normalized Enrichment Score
NK	Natural Killer
OS	Overall Survival
PD-1	Programmed Cell death protein 1
PDL1	Programmed Death Ligand 1
PET-TC	Positron emission tomography
PFS	Progression Free Survival
PI3K	Phosphoinositide 3-kinase
PRF	Perforin
TAP	Tapasin
TIL	Tumor Infiltrating Lymphocyte
TIS	T-cell Inflammatory Signature
TMB	Tumor Mutational Burden
TME	Tumor microenvironment
TNF-α	Tumor Necrosis Factor alpha
UM	Uveal Melanoma
VEGF	Vascular Endothelial Growth Factor
