# Supplementary figures and images for: Additive Role of Immune System Infiltration and Angiogenesis in Uveal Melanoma Progression
Source: Int J Mol Sci. 2021 Mar 6;22(5):2669. doi: 10.3390/ijms22052669 (PMC7961481; doi:10.3390/ijms22052669)

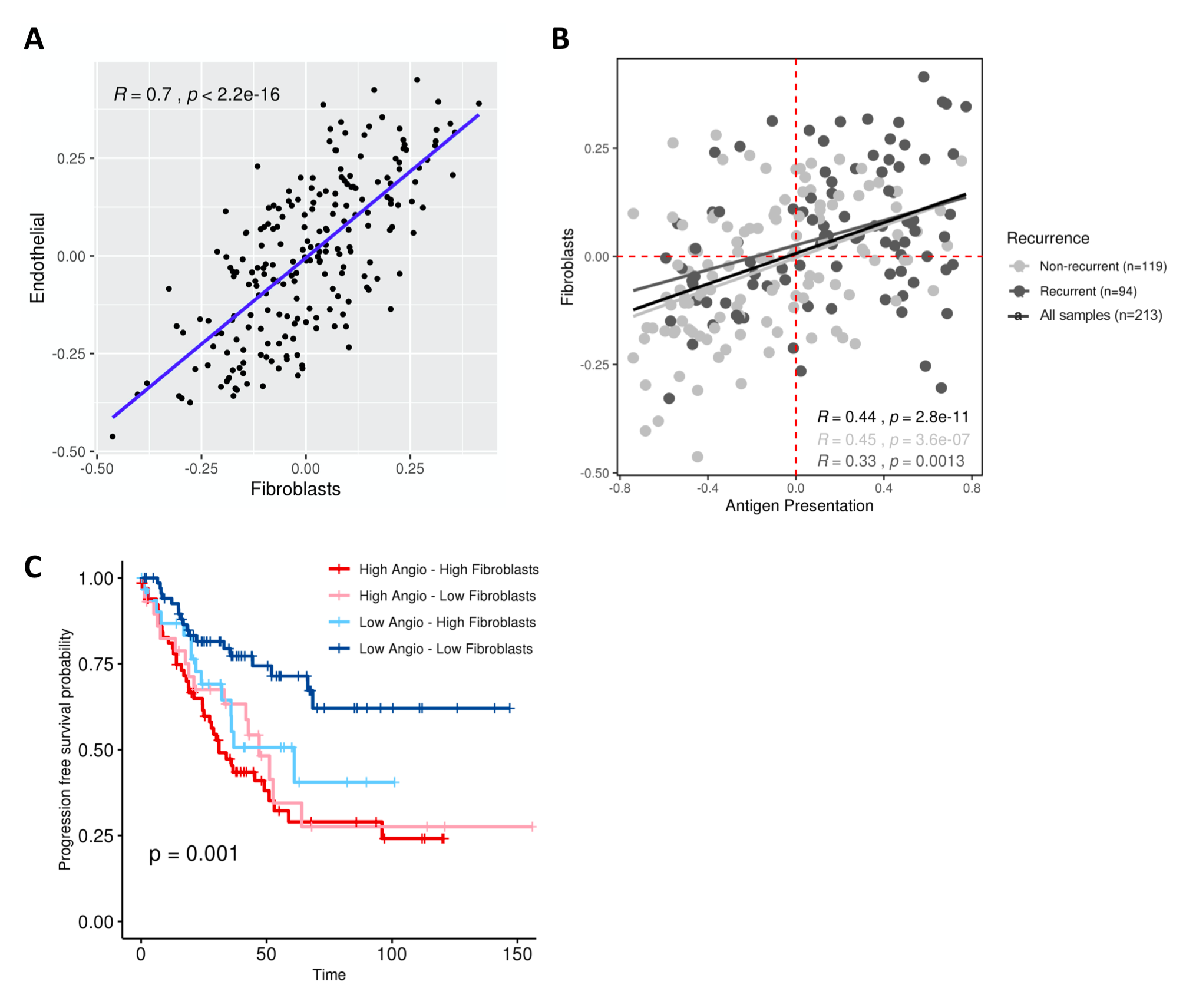

Supplement: Supplementary file 1 [file ijms-22-02669-s001.zip › Figure S6.tiff]

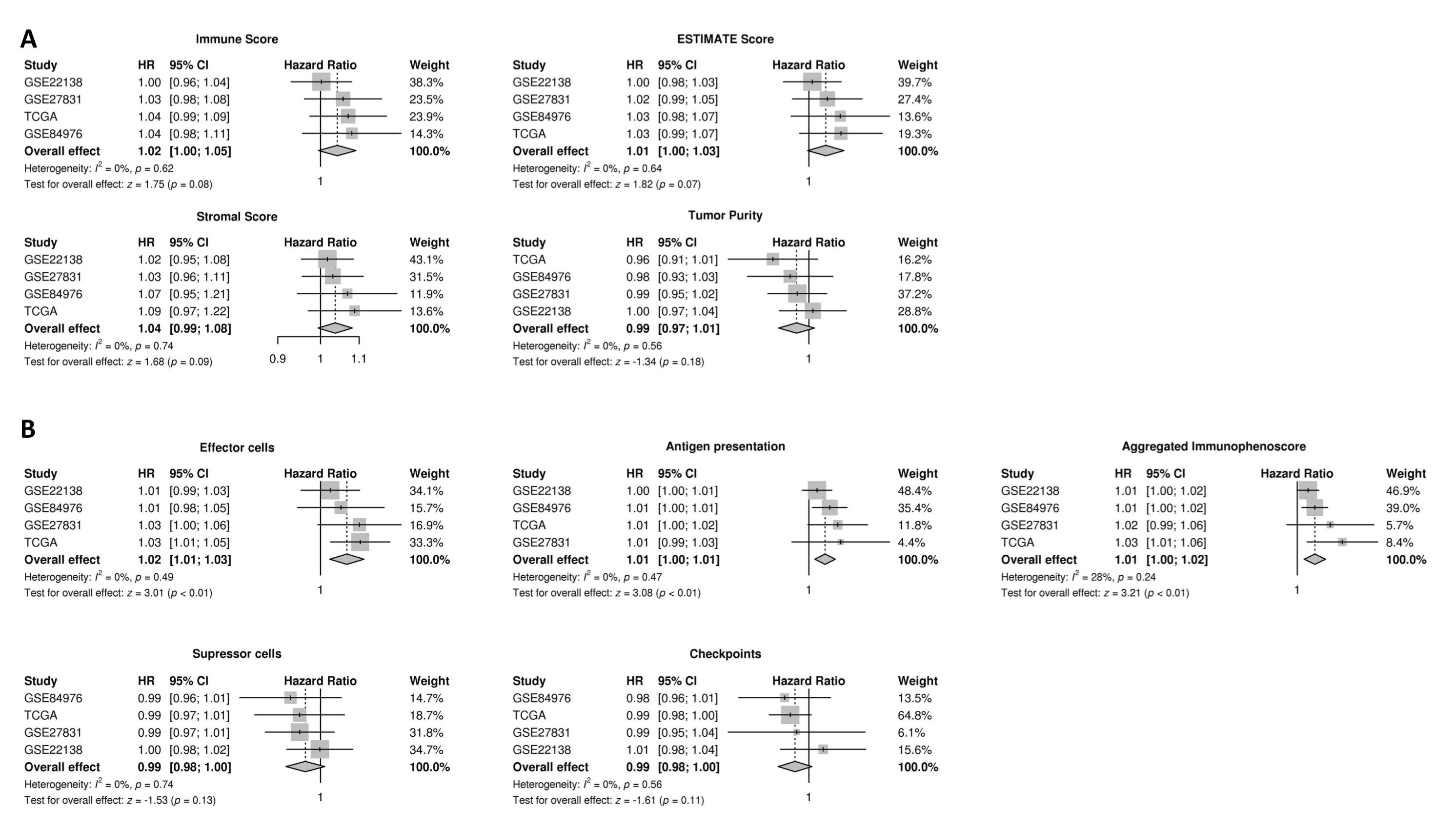

Supplement: Supplementary file 1 [file ijms-22-02669-s001.zip › Figure S1.tiff]

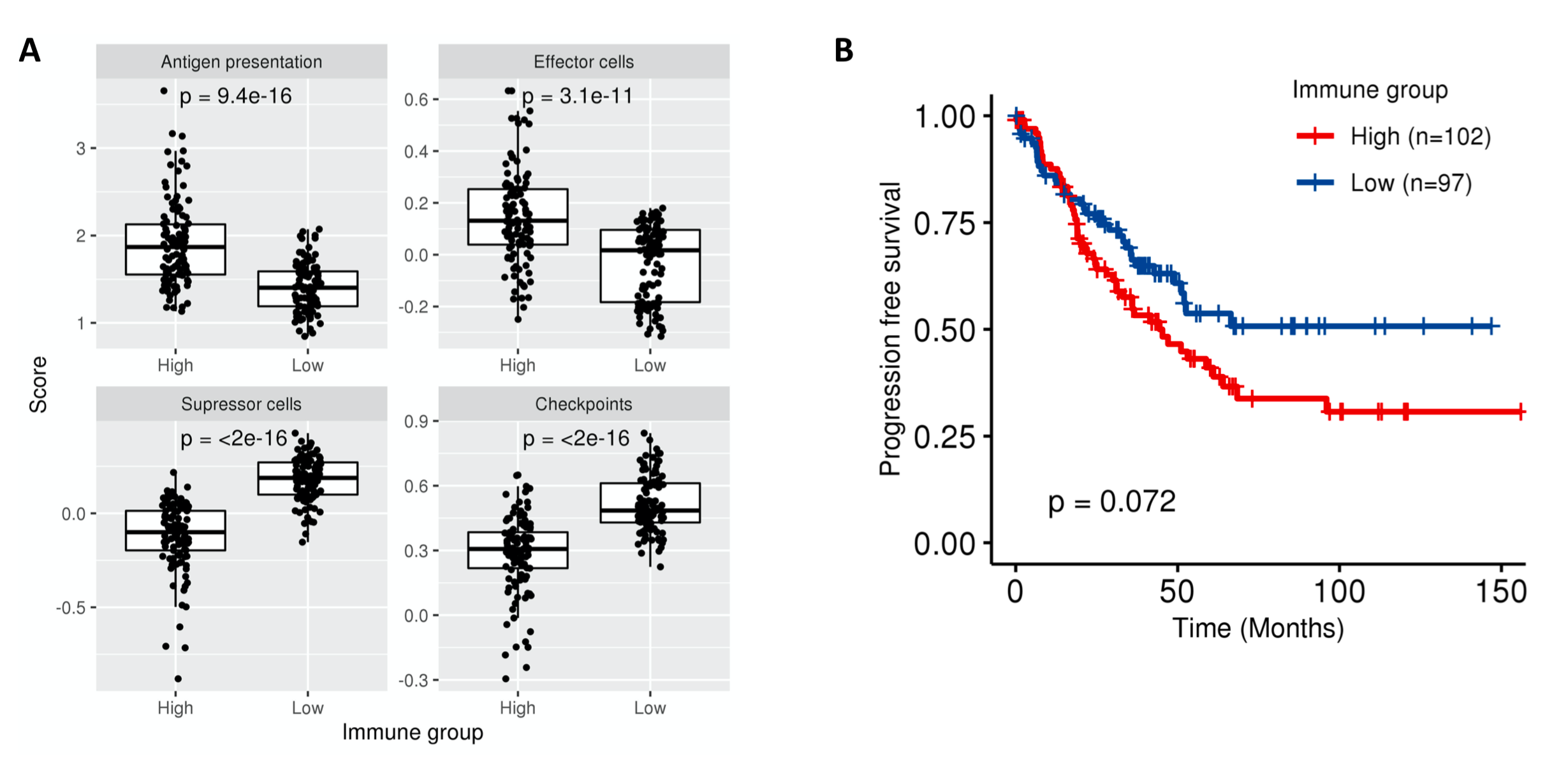

Supplement: Supplementary file 1 [file ijms-22-02669-s001.zip › Figure S2.tiff]

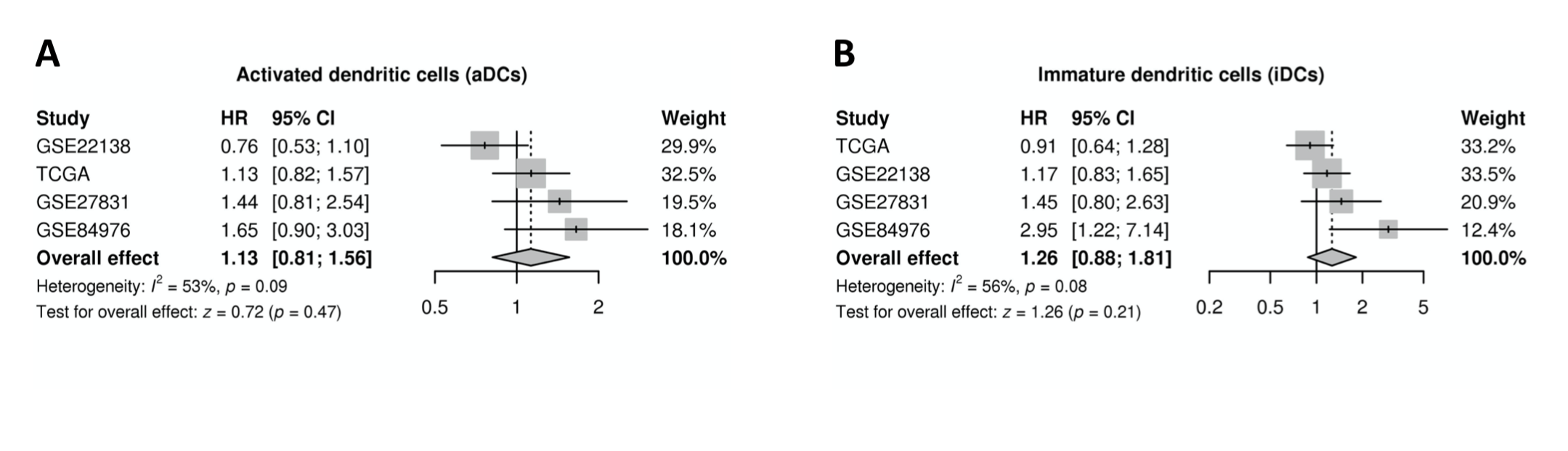

Supplement: Supplementary file 1 [file ijms-22-02669-s001.zip › Figure S3.tiff]

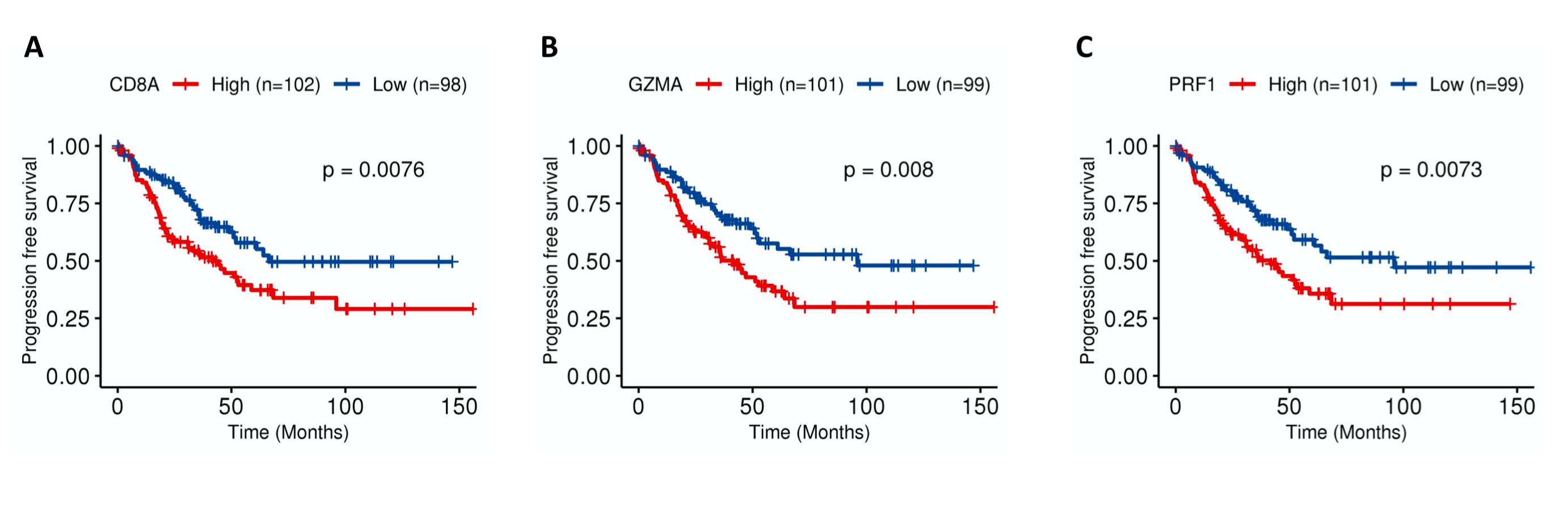

Supplement: Supplementary file 1 [file ijms-22-02669-s001.zip › Figure S4.tiff]

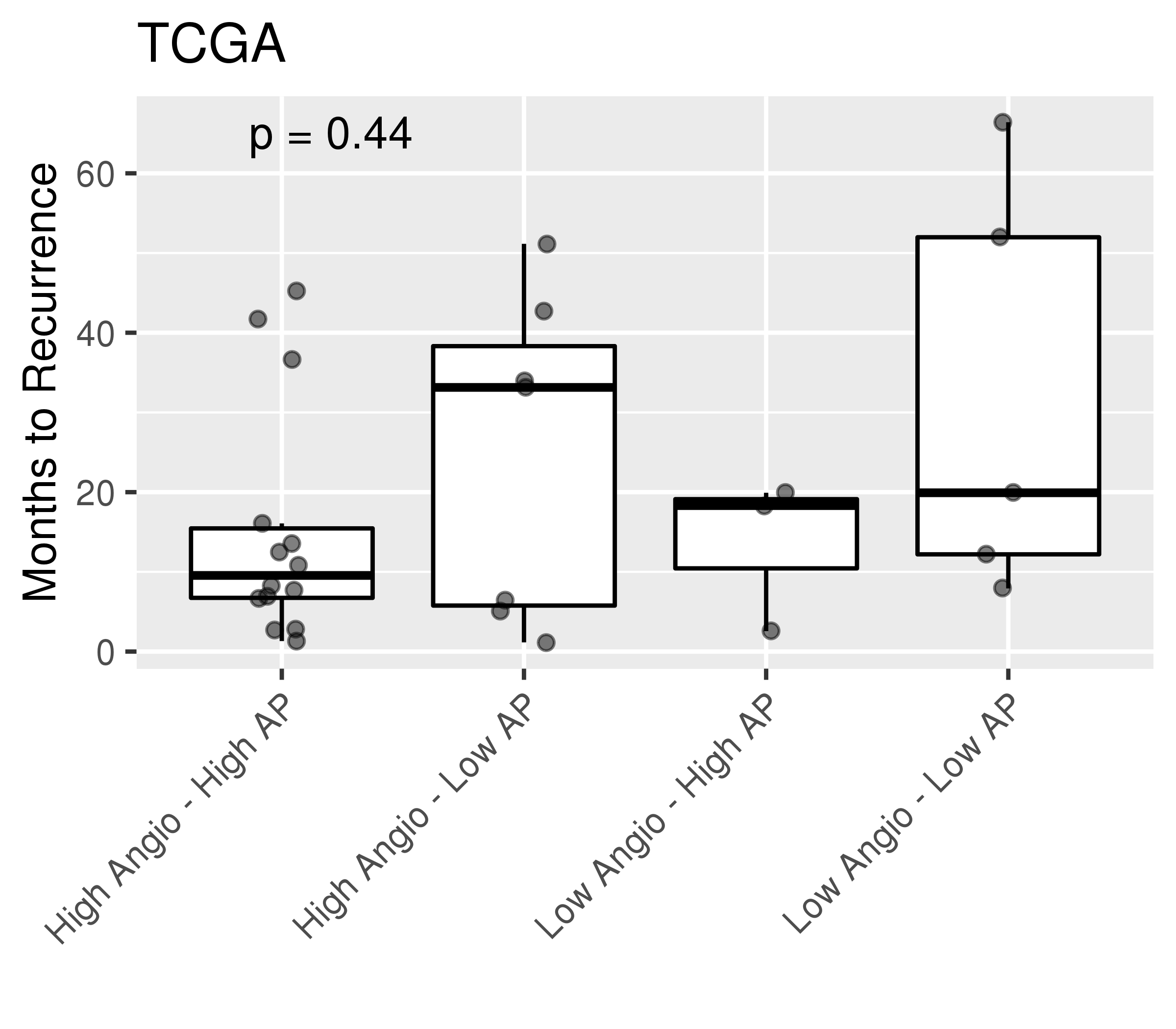

Supplement: Supplementary file 1 [file ijms-22-02669-s001.zip › Figure S5.tiff]
